# GutMicrobiotAware: an international exploratory survey on awareness and understanding of the gut microbiota

**DOI:** 10.3389/fmicb.2025.1643257

**Published:** 2026-01-09

**Authors:** Enriqueta Garcia-Gutierrez, Sara Arbulu, Charlotte Oliver, Sandeep Kumar, Sarita A. Dam, Babette Jakobi, Vincenzo Pennone, Fabiana A. Hoffmann Sarda, Arghya Mukherjee, Paul D. Cotter

**Affiliations:** 1 Teagasc Food Research Centre, Moorepark, Cork, Ireland; 2 APC Microbiome Ireland, University College Cork, Cork, Ireland; 3 VistaMilk SFI Research Centre, Moorepark, Cork, Ireland; 4 Department of Agronomic Engineering, Technical University of Cartagena, Cartagena, Spain; 5 Faculty of Chemistry, Biotechnology and Food Science, Norwegian University of Life Sciences, Ås, Norway; 6 Automation and Plant Engineering Division, ICAR-National Institute of Secondary Agriculture, Ranchi, India; 7 SeqBiome Ltd., Cork, Ireland; 8 Department of Human Genetics, Donders Institute for Brain, Cognition and Behaviour, Radboud University Nijmegen Medical Center, Nijmegen, Netherlands; 9 Cell and Tissue Engineering Laboratory, IRCCS Istituto Ortopedico Galeazzi, Milan, Italy; 10 Faculty of Science and Engineering, University of Limerick, Limerick, Ireland; 11 Health Research Institute, Limerick, Ireland

**Keywords:** dissemination, gut microbiome, gut microbiota, healthcare, science communication, survey

## Abstract

Over the past two decades, scientific understanding of the composition and function of the human gut microbiome has expanded substantially. The recent surge in human gut microbiota-related studies has unveiled the profound influence of the gut microbiota on host nutrition, health, and behavior, bridging biology, medicine, and ecology, among others. The dynamic interaction between daily lifestyle choices, life events, and the gut microbiota makes it understandably, a topic of interest among the lay public. Communicating scientific insights from the laboratory to the population effectively, however, can be challenging, and might involve adapting the delivery of knowledge to different audiences, using precise language in corresponding settings and the use of more accessible concepts in public forums such as science festivals or social media. With the growing interest in gut microbiota beyond academic circles, there is also an increased risk of disseminating information lacking scientific rigor. The current study aimed to assess the general knowledge regarding the gut microbiota among an exploratory pool of participants, primarily accessed via academic and social networks, and evaluate healthcare professionals’ understanding of its links to various health conditions, ultimately informing better communication strategies for both groups. Our findings from this exploratory survey indicate that while most participants were familiar with the gut microbiota, instances of partial and even complete misconceptions persisted. The results from our survey further underlined the need for targeted scientific communication to emphasize the microbial diversity of the gut microbiota, the factors influencing it, its links to health conditions, and the realistic scope of current microbiota analyses.

## 1 Introduction

Over the past two decades, the critical contribution of the human gut microbiota (the community of bacteria, archaea, viruses, fungi, and protozoa living in our gastrointestinal tract) to general human health, has become increasingly apparent (Yue et al., 2020; Prados-Bo and Casino, 2021). Disruptions in the gut microbiota or alterations in specific microbial signatures, known as dysbiosis, have been associated with various gastrointestinal conditions, such as inflammatory bowel disease (IBD), where alterations in the gut microbiota can lead to immune dysregulation and impaired intestinal barrier function (Afzaal et al., 2022). Obesity has additionally been associated with changes in the microbial diversity of the gut microbiota, with a dysbiotic impact recorded for members of the gut microbiota that enhance energy harvest from the diet and modulate host metabolism (Hu et al., 2024). Furthermore, in diabetes mellitus, gut dysbiosis contributes to impaired glucose metabolism and systemic inflammation (Garcia-Gutierrez et al., 2024). Neurological disorders, including Parkinson’s and Alzheimer’s diseases, have been linked to dysfunction of the gut-brain axis, where microbial metabolites produced by the gut microbiota and inflammatory processes impacted by said dysbiosis may influence neurodegeneration (Romano et al., 2021; Laske et al., 2022). Notably, autism spectrum disorder has been associated with distinct gut microbial profiles, potentially impacting behavior through immune and metabolic pathways (Su et al., 2024). Other examples include asthma and rheumatoid arthritis, which are thought to involve gut microbiota-driven systemic immune dysregulation (Frati et al., 2018). Importantly, disruptions in the gut microbiota have been implicated in cancer development, particularly colorectal cancer, brought about through dysbiotic modulation of inflammation and production of carcinogenic metabolites (Cheng et al., 2020). Understandably, the gut microbiome has emerged as a therapeutic target of interest, prompting efforts to develop tools to understand and modulate this ecosystem in ways that can translate into patient care. Of note, the close link between diet and the gut microbiota, and in turn health, has piqued interests beyond academia, reaching the general public, diverse demographic groups, and the healthcare sector. The field has consequently been the focus of considerable efforts relating to scientific dissemination and communication (Shan et al., 2019; Marcon et al., 2021).

Understandably, depending on which group is being addressed, different approaches, activities and language use are required. Usually, while disseminating within the scientific community implies use of precise, unambiguous language (e.g., scientific articles and conferences), conveying the same information to a lay audience requires accessible concepts and language (e.g., science festivals, podcasts or social media) (Torres-Salinas et al., 2024). For example, use of analogies, pop-culture references, storytelling-based approaches or even use of memes, might be an effective way of relaying core takeaways from high-level research in the lab to the general audience (Riser et al., 2020). This not only allows the scientific messaging to achieve increased population penetration but also boosts engagement, which is usually the primary objective of such exercises. Importantly, as for all scientific topics, it is essential to transmit insights about the gut microbiota rigorously to raise awareness and educate not only the public but also healthcare professionals, as they are crucial components of our healthcare systems.

Current efforts to develop tools for assessing gut microbiota as part of overall health, while no longer in its nascency, are still in progress, and routine analysis and interpretation of the gut microbiota at the patient level are not yet fully available to the general population (Lee et al., 2024). This is significant and is precipitated by variations in the gut microbiota with diet, lifestyle, geographical location, and other factors, making it difficult to establish a universal healthy gut microbiota standard (Risely, 2020; Shanahan et al., 2021; Pedroza Matute and Iyavoo, 2023). Furthermore, while there are various services claiming to analyse the gut microbiota, the quality and the accuracy of their claims vary, reflecting ongoing debates in the field. To fully harness the potential of the gut microbiota, it is necessary to address current knowledge gaps, particularly in understanding intra-microbial interactions and those between the microbes and their host. This includes gaining a deeper understanding of less studied organisms such as fungi and viruses, getting insights into microbial functions in addition to the descriptive composition, conducting large-scale temporal and spatial studies and using all this information to understand this complex ecosystem holistically (Shanahan et al., 2021). The current study, GutMicrobiotAware (or GMWare in short) aims to assess the general knowledge about the gut microbiota among an exploratory pool of international participants, most of whom were highly educated. Although exploratory in nature, we strived to make the survey as comprehensive as possible with questions gauging the general level of interest among responders, the sources of media responders would use to learn about the gut microbiota as well as the specific knowledge of the participants concerning related concepts. Additionally, we queried to what extent gut microbiota analyses are in routine practice among healthcare professionals. Ultimately, the information and insights generated in GMWare are minimally expected to (i) serve as a foundation to design more comprehensive and better informed surveys on the gut microbiota in the future, and (ii) configure more effective strategies to communicate gut microbiota research, both among scientific and lay audiences.

## 2 Methods

### 2.1 Questionnaire design

GMWare was conducted between March 2022 and November 2023, using an online questionnaire via the SurveyMonkey platform^1^. The questionnaire was designed by the authors and consisted of 22 or 23 questions (for versions meant for participants from the United States of America, India, and China, an additional question regarding province location was included) grouped in three blocks: (i) demographic information; (ii) general knowledge relating to the gut microbiota and its connections with human health and (iii) application of gut microbiota-related knowledge in the healthcare sector. The questionnaire was designed in English and translated intoBangla, Chinese, Dutch, French, German, Hindi, Italian, Portuguese, Punjabi and Spanish by native speakers who are also proficient in English. Answers were selected from multiple choice options and opt-outs like “I do not know,” “I am not X,” “I prefer not to respond” were provided as applicable as research suggests that including such options improves completion levels (Hu and Jerrod, 2020; Garcia-Gutierrez et al., 2023). Additionally, before releasing the final survey, a pilot trial was performed to ensure that the time to complete the survey was within the 5–6-min range, and that the questions were clear and understandable.

### 2.2 Participant recruitment

Participation was voluntary, independent and without compensation. The only exclusion criterion was age, as we only collected responses from participants ≥18 years old. Neither personal information nor IP addresses were collected, making the responses completely anonymous. The questionnaire was distributed via different channels: (i) university communities and networks of collaborators; (ii) social media and email lists of research funding bodies and other organizations; (iii) social media groups; (iv) family and friends’ networks.

### 2.3 Data visualization

To provide an overview of the survey responses, proportions were calculated for each response category. Given that participants could select multiple options, all analyses were performed at the response level rather than the respondent level. The resulting proportions were then visualized using R version 4.4.1 (2024-06-14) to illustrate trends across healthcare and non-healthcare participants. The R packages *rnaturalearth, rnaturalearthdata*, and *sf* were used to generate the participants distribution map. Answers were manually curated to identify potential inconsistencies within questionnaires (e.g., answering to healthcare answers if previously identified as non-healthcare workers). The final number of curated responses is indicated in each figure. Datasets generated and analyzed for this study can be found at https://github.com/sara-arag/Gut_MicrobiotAware_survey.

## 3 Results and discussions

### 3.1 Participant socio-demographic profile

**FIGURE 1:**
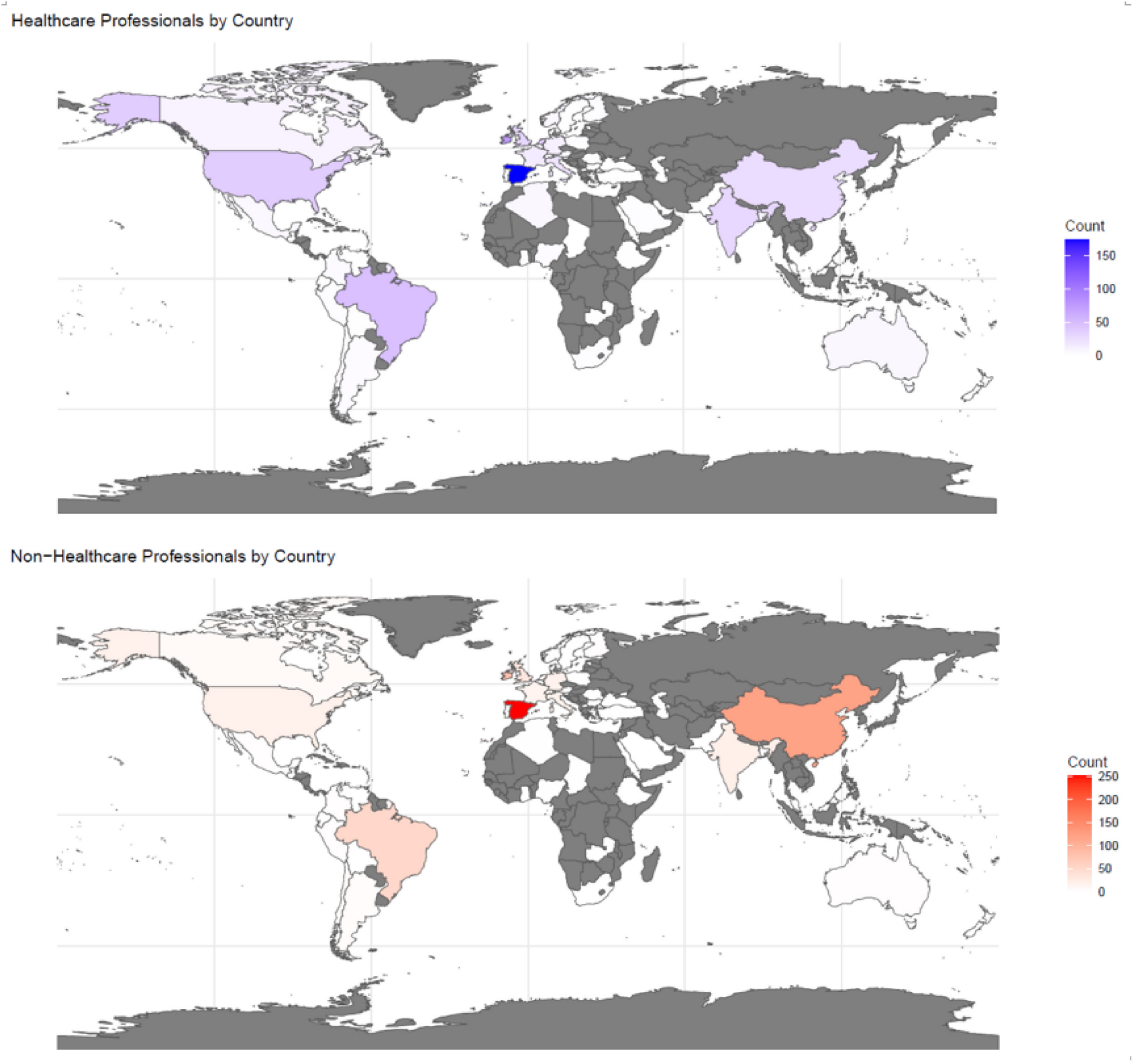
Participation in the GutMicrobiotAware study globally stratified by healthcare and non-healthcare participants. Countries shaded in purple represent healthcare professionals, countries shaded in red represent non-healthcare professionals, and countries colored in gray denote no participation.

A total of 1,288 participants from 54 countries across the five continents completed the survey between March 2022 and November 2023 (Figure 1). Spain was the country with the largest number of participants (425), followed by Ireland (151), China (144), and Brazil (98) (Supplementary Table 1). A total of 41 countries had less than 10 participants. This does not allow a deep study of the dissemination strategies in the different countries but provides an overview of how widespread certain microbiota concepts are. It is noted that there is an overrepresentation of the number of Ph.D., M.D. or similar-educated participants (29.36%). These numbers are influenced by the survey circulation channels that included universities and research centers, which resulted in a large percentage of participants having undertaken higher education. In a global context, Ph.D. holders only account for 1% of the world population (OECD, 2023), which sets the context for the education range. Note that healthcare-related jobs included clinical and research occupations that could be, but not necessarily, linked to the gut microbiota. For example, microbiologists are research scientists but not all of them work within the gut microbiota field and, therefore, might be less familiar with the topic.

Participants’ age ranged from 18 to over 50 years old with different educational backgrounds and professions were grouped as health- or non-health-related (Table 1). Students were categorized based on the field of their current studies.

**TABLE 1:** Socio-demographic characteristics of GutMicrobiotAware participants.

Characteristic	Options	Respondents	Percentage (%)
Age range	18–20	28	2.19
	21–29	361	28.22
	30–39	357	27.91
	40–49	253	19.78
	50–59	183	14.31
	60 or older	98	7.66
Highest level of education completed	Primary education	7	0.55
	High school	103	8.13
	Bachelor’s degree	344	27.15
	Master’s degree	373	29.44
	Ph.D., M.D. or similar	372	29.36
	Other	87	6.87
	Prefer not to say	13	1.03
Healthcare professional or scientist	Medical doctor general practitioner	3	0.33
	Medical doctor gastroenterologist	2	0.22
	Medical doctor neurologist	4	0.44
	Medical doctor specialist (other categories)	34	3.74
	Nurse	28	3.07
	Nutritionist	51	5.6
	Microbiologist	141	15.5
	Food technologist	54	5.93
	Pharmacist	31	3.4
	Veterinarian	14	1.53
	Other researcher/scientist	157	17.25
	Retired from any of the mentioned categories	6	0.66
	I was a healthcare professional or researcher but switched careers	18	1.32
	I prefer not to say	12	13.74
	Other	445	39
Not a healthcare professional or scientist	Architecture and engineering occupations	42	5.49
	Arts, design, entertainment, sports, and media occupations	30	3.92
	Business and financial occupations	35	4.57
	Cleaning and maintenance occupations	8	1.04
	Community and social service occupations	21	2.74
	Computer and mathematical occupations	64	8.37
	Construction and extraction occupations	2	0.26
	Education, training and library occupations	107	13.99
	Farming, fishing and forestry occupations	62	8.10
	Food preparation and serving related occupations	52	6.8
	Installation, maintenance and repair occupations	3	0.39
	Legal occupations	9	1.17
	Management occupations	33	4.31
	Office and administrative support occupations	61	7.97
	Personal care and service occupations	3	0.39
	Production occupations	9	1.17
	Protective service occupations	2	0.26
	Sales and related occupations	23	3
	Social science occupations	14	1.83
	Transportation and materials moving occupations	5	0.65
	Other	133	17.38
	Prefer not to say	47	6.14

Social demographic data were collected during the survey including data on age, highest level of education completed and association with healthcare related profession.

In the health-related group, “Microbiology” was the most reported occupation, along with researchers and scientists working in other areas (e.g., physicist, zoologist, entomologist, etc.) and other healthcare occupations (physiotherapist, ophthalmologist, etc.). Among the non-healthcare group, the most commonly reported occupations across all age groups were in food preparation, farming, fishing and forestry, and education, training, and library services.

### 3.2 Awareness and preferred information sources on the gut microbiota

Over the last two decades, the gut microbiota research field has become a rapidly evolving area of research in medical sciences, with important implications for human health (Gao et al., 2023). This new knowledge transcends the boundaries of the research community in the field to reach society at all levels. By including both healthcare and non-healthcare related professionals the survey gave the opportunity to compare the extent to which gut microbiota-related knowledge is spread among the non-healthcare professionals relative to healthcare professionals, the latter being increasingly important players for applying gut microbiota knowledge. The obtained results allowed us to identify knowledge gaps and misperceptions about the gut microbiota.

**FIGURE 2:**
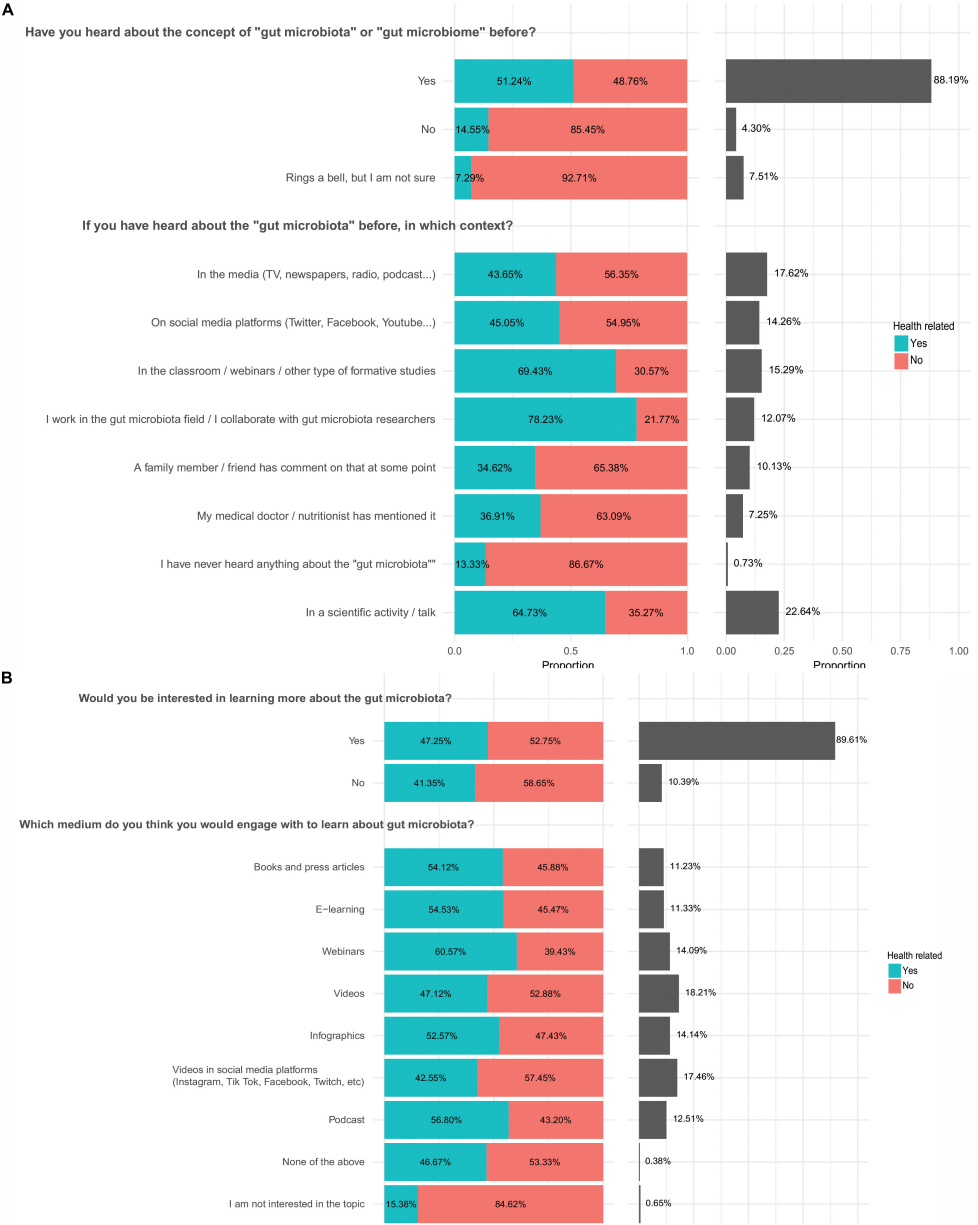
Sources of information and preferred learning channels on gut microbiota. **(a)** Sources of information used by the participants (number of responses for each question in order of appearance: *n* = 1,279 *n* = 996). **(b)** Sources of information the participants would be willing to use. The percentages indicate the proportion of answers relative to the total number of responses for each specific question (number of responses for each question in order of appearance *n* = 1,280, *n* = 1,255). The left panel shows the proportion of answers selected by healthcare and non-healthcare respondents. The right panel shows the overall proportion of all participants selecting each answer. The proportion of healthcare participants is shown in turquoise.

The majority of responses (88.19%) reported being familiar with the term “gut microbiota,” while 4.30% had not heard of it, and 7.51% were unsure (Figure 2a). The proportion of “Yes” responses was similar between health-related (51.24%) and non-health-related respondents (48.76%). However, among responses indicating uncertainty about the term, the proportion of non-health-related responses was higher (85.45%) (Figure 2a). Where relevant, when the participants were asked where they had heard about the gut microbiota concept, scientific activities/talks was the most selected answer (22.64%), with a higher proportion of these responses coming from health-related participants (64.73%). All the other options, including social media, health advisors, training and education or word of mouth with family and friends, ranged from 7.25 % to 17.62% of the answers, indicating the potential importance of using multiple different routes to disseminate information (Figure 2a). These results are in agreement with the International Microbiota Observatory survey 2024 that reported 70% of the participants had heard about the microbiome term mainly via healthcare professionals, schools, and TV shows (Biocodex Microbiota Institute, 2024) (note that the terms microbiota (collection of microbes) and microbiome (collection of microbes and their genes) are used interchangeably in some of these studies (Ursell et al., 2012)).

Participants learnt about gut microbiota and wanted to continue learning using a variety of information channels, highlighting their interest in the topic and multiple options to deliver effective gut microbiota research outreach, such as videos, social media platforms, infographics, webinars or podcasts. Figure 2b shows the most commonly used media through which individuals would be interested in learning about the gut microbiota. Videos (18.21%) and videos on social media platforms (17.46%) were the most selected options, followed by infographics (14.14%), while books and press articles were the least popular selection (11.23%). Webinars (14.09%), podcasts (12.51%) and e-learning (11.33%) were other alternatives that respondents would choose to learn about the gut microbiota. Both health and non-health-related participants shared similar rates of affinity for the different options (Figure 2b). When asked if they previously searched for gut microbiota information and which were their information sources, participants selected research articles (49.28%) and social media (15.95%) as the most previously used channels (Supplementary Figure 1). This aligns with findings from Abu-Humaidan, who reported that participants preferred learning through social media and trusted sources (Abu-Humaidan et al., 2021).

### 3.3 Understanding of gut microbiota concepts and perceived health links

**FIGURE 3:**
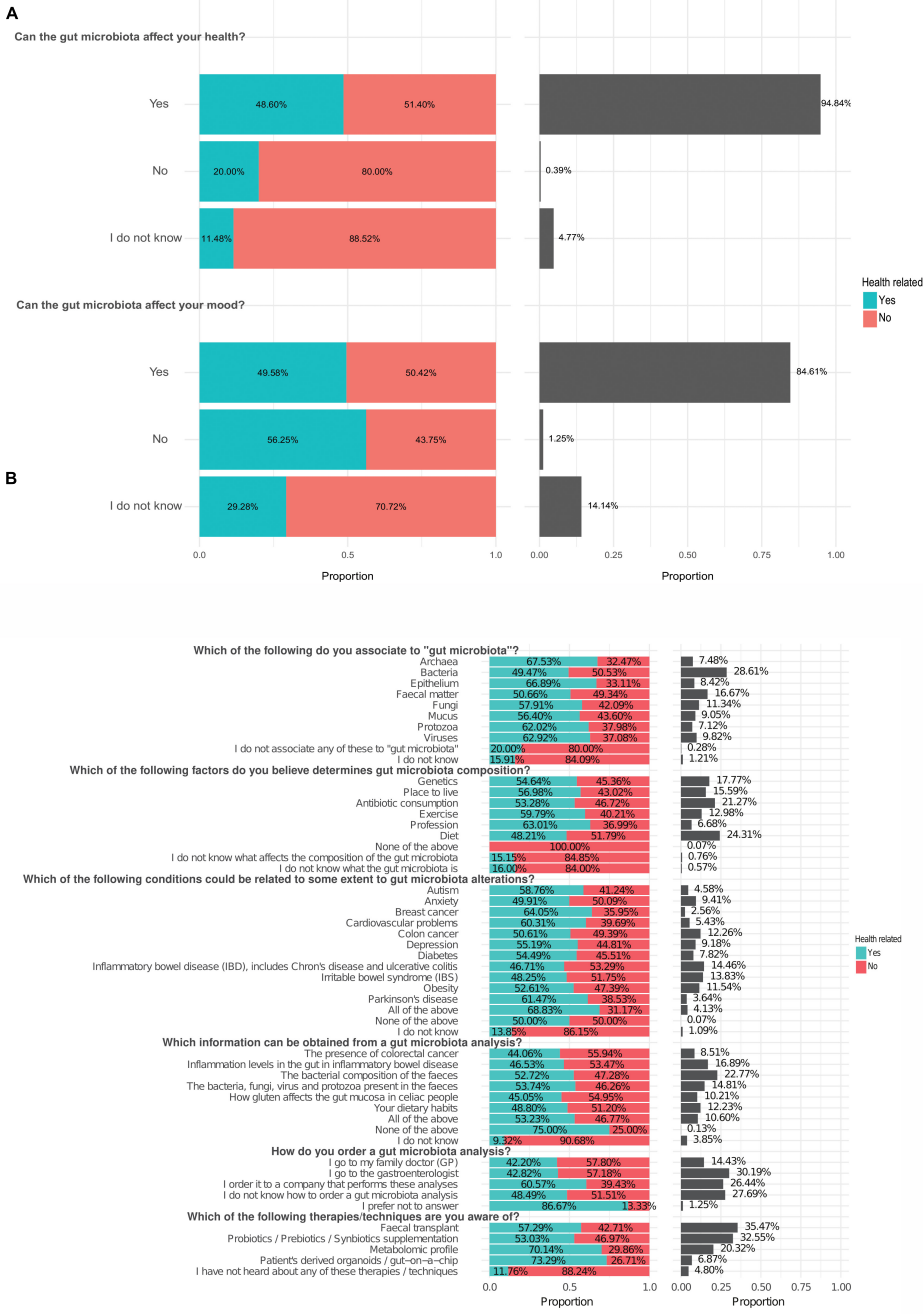
Gut microbiota-related concepts. **(a)** Participants’ perceptions of whether the gut microbiota affects health and mood (*n* = 1,280, *n* = 1,280). **(b)** General knowledge of gut microbiota-related concepts. The percentages indicate the proportion of answers relative to the total number of responses for each specific question. The left panel shows the proportion of answers selected by healthcare and non-healthcare respondents. The right panel shows the overall proportion of all participants selecting each answer. The proportion of healthcare participants is shown in turquoise.

To begin exploring the general knowledge vis-à-vis the gut microbiota, participants were asked whether they believed the gut microbiota affects overall health and mood (Supplementary Table 2). The vast majority answered positively in both cases (94.84% and 84.61% of the reponses, respectively) (Figure 3a), indicating a broad recognition of its potential relevance beyond digestion. To further assess the depth of this knowledge, six questions pertaining to terms and concepts relating to gut microbiota and health were included in the survey (Figure 3b). Participants associated “Bacteria” with gut microbiota in 28.61% of the responses. This is not surprising since the bacteriome is by far the best characterized component of the gut microbiota (Vemuri et al., 2020), while viruses, fungi, archaea and eukaryotic organisms are now slowly gaining more attention (Matijašić et al., 2020; Pargin et al., 2023). The second concept that was most associated with gut microbiota was “Fecal matter” (16.67%). Most of the gut microbiota composition analysis are performed using fecal samples due to their easy collection and processing. Importantly, while fecal matter serves as an indicator of gut microbiota composition, it is not representative of all sub-habitats along the gastrointestinal tract, access to which requires more invasive and costly biopsy-like procedures (Tang et al., 2020) or newer gut content-capturing capsule technologies (Waimin et al., 2020; Nejati et al., 2022). A closer observation revealed similar levels of acquaintance with the terms in the two groups. Among healthcare professionals, 49.47% and 50.66% of responses recognized the terms “Bacteria” and “Fecal matter”, respectively, while 50.53% and 49.34% of non-healthcare professionals responses recognized the same terms (Figure 3b). The remaining concepts were more selected by health-care related participants. Those included “Fungi” (11.34%), closely followed by “Mucus” (9.05%), “Epithelium” (8.42%) and “Viruses” (9.82%). “Protozoa” (7.12%) and “Archaea” (7.48%) were less associated with gut microbiota. A small percentage of responses accounted for not knowing (1.21%) or did not associate any of these terms with the gut microbiota (0.28%), most of those being from non-health-related occupations (Figure 3b). Knowledge about the composition, factors influencing the gut microbiota, and related conditions were therefore mainly associated with concepts or conditions directly related to the gastrointestinal tract, which was also observed in a gut microbiota survey targeted at dietitians (Mitsou et al., 2024).

Among the factors that affect gut microbiota composition, diet (24.31%) was the most frequently selected factor. The proportion of responses was similar between healthcare-related (48.21%) and non-health-related (51.79%) participants. Other factors were selected in a higher proportion by healthcare participants and were less associated to the gut microbiota by non-healthcare participants (Figure 3b): antibiotic consumption (21.27%) were the most reported ones, while others like genetics (17.77%), place to live (15.59%), exercise (12.98%) and profession (6.68%) were less selected. In the opt-out options, a small percentage of primarily non-health responses reported not knowing what affects the composition of the gut microbiota (0.76%) or not knowing what the gut microbiota is (0.57%). Only 0.07%, exclusively from non-health-related participants, selected that none of the factors affected the gut microbiota. Notably, diet and antibiotics were regarded as the two main factors that influence the gut microbiota, which is consistent with a previous survey in which 76% and 67% of the participants also selected these two factors, respectively, as being of key importance (Biocodex Microbiota Institute, 2024). Another survey circulated among students in Jordan showed that 91.3% of the participants agreed that antibiotics impact beneficial bacteria (Abu-Humaidan et al., 2021). Research has shown that low microbiota diversity is associated with inflammatory diseases and metabolic disorders (Rinninella et al., 2023), while eating a wide variety of foods, especially fermented foods and fiber-rich and an exercise lifestyle supports a healthy gut microbiota (O’Sullivan et al., 2015; Boytar et al., 2023; Chen et al., 2023; Mukherjee et al., 2024; Ross et al., 2024). In addition, numerous campaigns have drawn attention to the antimicrobial resistance crisis and the appropriate use of antibiotics (Davis et al., 2018; Will, 2020). Antibiotics can directly alter the gut microbiota composition, decreasing its diversity (Patangia et al., 2022). Other factors such as genetics, place of living, exercise or profession were selected, highlighting a good knowledge of the variety of factors that can influence the gut microbiota among the participants. In the study by Abu-Humaidan et al. (2021) participants also considered that the exercise affects the gut microbiota.

When participants were asked which conditions they believed were associated with the gut microbiota, conditions such as inflammatory bowel disease (IBD), including Crohn’s disease and ulcerative colitis (14.46%), irritable bowel syndrome (IBS) (13.83%), and colon cancer (12.26%) were among those most frequently selected, while conditions that were not directly connected with the gut, like cardiovascular problems (5.43%), Parkinson’s disease (3.64%) and breast cancer (2.56%) were less associated with the gut microbiota (Figure 3b). Systemic conditions like obesity (11.54%) and diabetes (7.82%) were selected to a similar degree as conditions associated with the brain/nervous system such as anxiety (9.41%) and depression (9.18%), whereas autism (4.58%) was not as perceived to be as highly associated with the gut microbiota. A total of 4.13% of responses identified all conditions as being associated to some extent with the gut microbiota, and only 0.07% indicated no association for any of the listed conditions. Non-healthcare respondents more frequently selected “I do not know” (1.09%). Thus, the conditions regarded as being related to the gut microbiota were those associated with gastrointestinal health, e.g., IBD, IBS, colon cancer or obesity. Awareness of areas where an increasing amount of research is being performed, such as those relating to the gut-brain axis, could benefit from continued science communication.

We were also interested in learning what participants perceived they would be provided with through a gut microbiota analysis (Figure 3b). Their view was that such tests can give information on various gut-related conditions. The most selected option was the bacterial composition of the feces (22.77%), although other options that were frequently selected were: inflammation levels in the gut in inflammatory bowel disease (16.89%), how gluten affects the gut mucosa in celiac people (10.21%), inform of the presence of colorectal cancer (8.51%) and dietary habits (12.23%). Moreover, 10.60% of responses indicated that such an analysis could provide information of relevance across all of these areas, while only 0.13% indicated that it could not provide any of these insights. The opt-out option “I do not know” option was selected in 3.85% of responses from non-healthcare participants.

The use of gut microbiota analysis is not widespread in clinical practice, and this was reflected in the participants’ answers. When asked how to order a gut microbiota analysis, visiting a gastroenterologist (30.19%) or going to their family doctor (14.43%) were more selected by non-healthcare participants (57.18% and 57.80%, respectively), while ordering it from a private company accounted for 26.44% of the responses (60.57% health-care participants, 39.43% non-healthcare participants, respectively) (Figure 3b). Not knowing how to order a gut microbiota analysis was selected in 27.69% of the recorded responses.

The last question of this block referred to therapeutic options that are currently under research or have recently started to be available for patient care (Figure 3b). The answers of the respondents were as follows: fecal transplants (39.02%) (Chen and Chiu, 2022), probiotics (Wang et al., 2024), prebiotics and synbiotic supplementations (26.57%), metabolomic profiling (21.76%), and organoids and gut-on-a-chip technology (4.80%). These results demonstrate an awareness of these options and open opportunities to further educate on the advantages and disadvantages of different modulation strategies as the supporting science continues to develop.

### 3.4 Integration of gut microbiota analysis in healthcare practice

**FIGURE 4:**
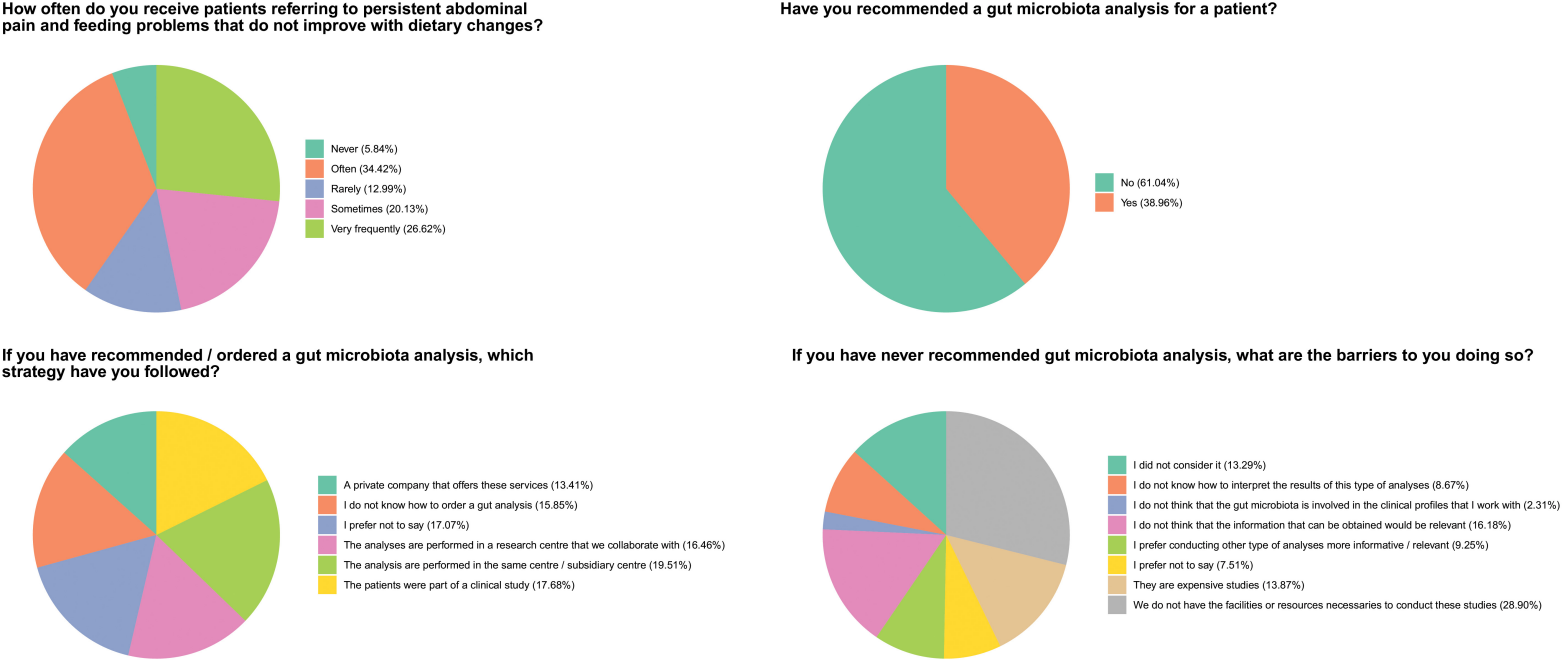
Healthcare professionals’ tendency toward gut microbiota analysis. As part of GutMicrobiotAware, healthcare professionals were queried about their preferences regarding gut microbiota analyses. The percentages indicate the proportion of answers relative to the total number of responses for each specific question (number of responses for each question in order of appearance *n* = 154, *n* = 154 *n* = 95 *n* = 107).

This block of four questions was designed to assess whether microbiota awareness is translated into medical practice (Figure 4). Each question in this block was answered independently, and responses were not conditional on previous answers.

Our survey suggests that some healthcare professionals “very frequently” (26.62%) or “often” (34.42%) receive patients who suffer from persistent abdominal pain and feeding problems that do not improve with dietary changes. About 38.96% of responses from the surveyed healthcare professionals recommended gut microbiota analyses, while 61.04% did not. Among the responses that supported recommending such analyses, 19.51% referred patients to the center where the healthcare professional was based or to a subsidiary center, and 17.68% did so as part of a clinical study. These results suggest that the gut microbiota studies are conducted on the basis of opportunity, i.e., due to availability of resources and/or collaborators with resources. Additionally, 16.46% of responses indicated referrals through a collaborating research center, while 13.41% indicated the use of a private company. Moreover, from the 61.04% of the healthcare workers who reported that they had never recommended a gut microbiota analysis, 51.44% of the responses indicated some level of resource limitation, facilities (28.90%), economic (13.87%) or knowledge-related (8.67%) issues

It is indicative that 38.96% of the healthcare participants did not recommend gut microbiota analyses or did not considered it (13.29%), highlighting a potential area of intervention with informative purposes. The debate around gut microbiota testing involves several key issues, reflecting both the promise and limitations of this emerging field (Qian and Ho, 2020). Accuracy and reliability of gut microbiota tests, especially at-home-kits, can lead to varying results from the same stool sample, suggesting that current testing technologies may not provide an accurate snapshot of an individual’s microbiome health (Servetas et al., 2024). Additionally, being the gut microbiota highly dynamic and influenced by diet, sleep, and stress (Gubert et al., 2020; Qi et al., 2022), rapid changes can result in tests becoming quickly irrelevant in a specific health context. The unique nature of each person’s microbiota further complicates the matter, making it difficult to define a “normal” gut microbiota (Qian and Ho, 2020). Furthermore, the lack of standardization in testing methodologies results in inconsistent outcomes (Vandeputte et al., 2017; Attaye et al., 2022), limiting their utility in clinical practice. While microbiome tests hold promises for diagnosing specific conditions, their application in general healthcare remains limited, as they often provide little actionable information for diagnosis or treatment requiring highly specialized and microbiota-trained health professionals. Because of this, there is a risk of misdiagnosis leading to inappropriate interventions and potential misuse of gut microbiome tests that offer unsupported health claims.

### 3.5 Misconceptions and future communication strategies

During the analysis of the survey results, several areas where misconceptions may arise were identified, highlighting the need to prioritize communication efforts to clarify these key concepts both in the health and non-health-worker environments. For instance, to increase low awareness of non-bacterial gut members, communication materials that visualize the diversity of microorganisms within the gut ecosystem, for example, “Meet your microbes” micro-profiles or mini-animations, could help shift perception from a bacteria-centric view to a more accurate ecological understanding.

The gut microbiota’s role in the overall health state was associated with gut-related issues by health-related and non-health-related respondents, while conditions like breast cancer, Parkinson’s disease or cardiovascular problems were less likely to be associated. It would be important to understand that the impact of the gut microbiota is not limited to the gut, but it extends to the whole human body in complex ways, intertwined with the immune system. Based on communication preferences acknowledged in this survey by both groups, we propose that campaigns of short videos, “knowledge pills” or “learning nuggets,” and infographics that can be easily shared on social media, are used to inform the public about the importance of maintaining a healthy gut microbiota not only to prevent gut issues, but as a keystone of overall health. For health-related professionals, webinars that can be attended in their own time, potentially with a system of microcredentials, could keep them informed of the advances in the gut microbiota research area, including new links between the gut and distant organs and new analytical and therapeutic options for personalized medicine, like organoids or metabolic profiling. Additionally, effective communication relating to the gut microbiota can educate healthcare professionals and broaden the repertoire of tools at their disposal to tackle patients’ conditions from a more holistic perspective, foster networking between scientists and the health system and educate the general public, while empowering the population to take control of their health. To achieve this, citizen science initiatives (Garcia et al., 2023; Walsh et al., 2024) and interdisciplinary approaches between the science community, the health systems, public engagement agents and governmental administrations are needed to design effective strategies towards a more educated society (Schelkle and Galland, 2020).

Misconceptions about the capabilities of gut microbiota tests (e.g., that they can diagnose colorectal cancer, inflammation levels, or coeliac disease) highlight the need for messaging that clearly differentiates between realistically actionable insights, like relative bacterial composition, and what is still under scientific development. Short explanatory modules for healthcare professionals, potentially integrated into continuing education formats, could support appropriate test use and patient guidance.

Another key concept to emphasize through information campaigns is the impact of antibiotics on gut microbiota, particularly during winter when demand for antibiotics often rises to treat infectious conditions. Regular reminders and clear explanations that not all infections require antibiotics, along with the potential impact of frequent antibiotic use on gut microbiota, could significantly enhance public awareness. Aligning with observations by Kokkinias et al. (2024), appealing to people’s values and emotions and using humor could be powerful strategies to communicate these scientific concepts to a wider audience. Therefore, using storytelling to connect gut microbiota concepts to personal and everyday experiences and health journeys can enhance relatability and capture attention. Additionally, using gamification such as interactive quizzes or games, can make these concepts more accessible and foster public engagement and retention of knowledge.

The data collected from this survey can guide future research in multiple directions, such as designing targeted educational interventions and evaluating diverse communication strategies. This approach could help assess the effectiveness of various science communication methods and identify best practices for disseminating specific concepts. For instance, engaging elderly individuals may require in-person meetings, while teenagers might respond better to digital tools.

## 4 Conclusion

In summary, this survey collected data on gut microbiota-related topics and the sources through which this information is accessed. It was clear that most respondents knew about the gut microbiota but misconceptions or incomplete knowledge was also present, highlighting areas that could be addressed through science communication and dissemination. These include emphasizing the multi-microorganism composition of the gut microbiota, factors that influence their composition, the various conditions associated with it, and more accurate and realistic information on gut microbiota tests and current modulation strategies.

## Data Availability

The datasets presented in this study can be found in online repositories. The names of the repository/repositories and accession number(s) can be found below: https://github.com/sara-arag/Gut_MicrobiotAware_survey.

## Footnotes

1
https://www.surveymonkey.com
