# Supplementary material for: GutMicrobiotAware: an international exploratory survey on awareness and understanding of the gut microbiota
Source: Front Microbiol. 2026 Jan 9;16:1643257. doi: 10.3389/fmicb.2025.1643257 (PMC12827544; doi:10.3389/fmicb.2025.1643257)
Supplement: Supplementary file 1 [file Data_Sheet_1.docx]

Supplementary Material

**GutMicrobiotAware: An International Exploratory Survey on Awareness and Understanding of the Gut Microbiota**

Enriqueta Garcia-Gutierrez*^#1,2,3,4^, Sara Arbulu^#5^, Charlotte Oliver^δ,1^, Sandeep Kumar^δ,6^, Sarita A. Dam^δ,7^, Babette Jakobi^δ,8^, Vincenzo Pennone^δ,9^, Fabiana A. Hoffmann Sarda^δ,10,11^, Arghya Mukherjee^1,2,3^ and Paul D. Cotter*^1,2,3^

^1^Teagasc Food Research Centre, Moorepark, Fermoy, Co. Cork, Ireland.

^2^APC Microbiome Ireland, University College Cork, Cork, Ireland.

^3^VistaMilk SFI Research Centre, Moorepark, Fermoy, Cork, Ireland

^4^Agronomic Engineering Department, Technical University of Cartagena, Cartagena, Spain

^5^Faculty of Chemistry, Biotechnology and Food Science, Norwegian University of Life Sciences, Ås, Norway.

^6^Automation and Plant Engineering Division, ICAR-National Institute of Secondary Agriculture, Ranchi, Jharkhand, India

^7^SeqBiome Ltd., Cork, Ireland

^8^Department of Human Genetics, Donders Institute for Brain, Cognition and Behaviour, Radboud University Nijmegen Medical Center, Nijmegen, The Netherlands

^9^Cell and Tissue Engineering Laboratory, IRCCS Istituto Ortopedico Galeazzi, via Cristina Belgioioso Milan, Italy

^10^Faculty of Science & Engineering, University of Limerick, Limerick, Ireland

^11^Health Research Institute, Limerick, Ireland

^#^These authors contributed equally

^δ^ These authors contributed equally

*** Correspondence:** Correspondence should be addressed to Enriqueta Garcia-Gutierrez([enriqueta.garcia@upct.es](mailto:enriqueta.garcia@upct.es)) and Paul D. Cotter ([paul.cotter@teagasc.ie](mailto:paul.cotter@teagasc.ie))

**Supplementary Table S1.** Number of participants in GutMicrobioAware stratified by nationality.

| **Country of origin** | **Number of participants** |  | **Health-related** | |
| --- | --- | --- | --- | --- |
|  |  | **Percentage of participants** | **Yes** | **No** |
| Algeria | 7 | 0.54 | 6 | 1 |
| Andorra | 1 | 0.08 | 1 | 0 |
| Anguilla | 1 | 0.08 | 0 | 1 |
| Argentina | 7 | 0.54 | 3 | 4 |
| Australia | 8 | 0.62 | 6 | 2 |
| Austria | 4 | 0.31 | 4 | 0 |
| Bangladesh | 7 | 0.54 | 7 | 0 |
| Belgium | 8 | 0.62 | 7 | 1 |
| Bolivia | 1 | 0.08 | 1 | 0 |
| Brazil | 98 | 7.61 | 47 | 51 |
| Bulgaria | 1 | 0.08 | 1 | 0 |
| Canada | 16 | 1.24 | 9 | 7 |
| Chad | 1 | 0.08 | 0 | 1 |
| Chile | 8 | 0.62 | 3 | 5 |
| China | 144 | 11.18 | 25 | 119 |
| Colombia | 6 | 0.47 | 4 | 2 |
| Czech Republic | 2 | 0.16 | 1 | 1 |
| Denmark | 4 | 0.31 | 4 | 0 |
| Ecuador | 3 | 0.23 | 1 | 2 |
| Ethiopia | 1 | 0.08 | 1 | 0 |
| Finland | 4 | 0.31 | 2 | 2 |
| France | 29 | 2.25 | 15 | 14 |
| Germany | 28 | 2.17 | 11 | 17 |
| Ghana | 2 | 0.16 | 2 | 0 |
| Guatemala | 2 | 0.16 | 0 | 2 |
| India | 49 | 3.80 | 29 | 20 |
| Ireland | 151 | 11.72 | 74 | 77 |
| Israel | 2 | 0.16 | 1 | 1 |
| Italy | 35 | 2.72 | 19 | 16 |
| Malaysia | 2 | 0.16 | 1 | 1 |
| Mexico | 7 | 0.08 | 5 | 2 |
| Netherlands | 47 | 3.65 | 37 | 10 |
| New Zealand | 4 | 0.31 | 2 | 2 |
| Nigeria | 4 | 0.31 | 3 | 1 |
| Norway | 7 | 0.54 | 5 | 2 |
| Pakistan | 1 | 0.08 | 1 | 0 |
| Palau | 1 | 0.08 | 0 | 1 |
| Peru | 4 | 0.31 | 2 | 2 |
| Poland | 3 | 0.23 | 2 | 1 |
| Portugal | 4 | 0.31 | 3 | 1 |
| Romania | 1 | 0.08 | 1 | 0 |
| Saudi Arabia | 2 | 0.16 | 2 | 0 |
| Singapore | 1 | 0.08 | 0 | 1 |
| Slovenia | 1 | 0.08 | 1 | 0 |
| South Africa | 2 | 0.16 | 2 | 0 |
| Spain | 425 | 33 | 174 | 251 |
| Sweden | 2 | 0.16 | 1 | 1 |
| Switzerland | 20 | 1.55 | 5 | 15 |
| Turkey | 2 | 0.16 | 1 | 1 |
| United Kingdom | 60 | 4.66 | 29 | 31 |
| United States | 55 | 4.27 | 37 | 18 |
| Venezuela | 1 | 0.08 | 1 | 0 |
| Zambia | 1 | 0.08 | 1 | 0 |

**Supplementary Table S2.** The GutMicrobiotAware survey. The questionnaire for the survey (English only) is presented here in a tabular format along with the answering options made available during the survey. The queries are marked in light blue (Q1 to Q4 are missing as they were related to consent, country and province or state depending on the country). The version presented below is in the minimal text format and may not necessarily tally exactly with the questionnaire description in the main text; some follow up questions are numbered simply as a different question due to the presence or absence of the province/state question. Translated questions in other languages are available at: <https://github.com/sara-arag/Gut_MicrobiotAware_survey>

| **Q5. What is your age range?** |
| --- |
| 18-20 |
| 21-29 |
| 30-39 |
| 40-49 |
| 50-59 |
| 60 or older |
| **Q6. What is the highest level of education you have completed?** |
| Primary education |
| High School |
| Bachelor's degree |
| Master's degree |
| PhD, MD or similar |
| I prefer not to say |
| Other (please specify) |
| **Q7. Are you a healthcare professional (medical doctor, nurse, nutritionist, pharmacist, veterinary...) or work in research in health-related topics?** |
| Yes |
| No |
| **Q8. If you have selected "healthcare" or "research", could you please specify?** |
| Medical doctor General Practitioner |
| Medical doctor Gastroenterologist |
| Medical doctor Neurologist |
| Medical doctor specialist (other categories) |
| Nurse |
| Nutritionist |
| Microbiologist |
| Food technologist |
| Pharmacist |
| Veterinarian |
| Other researcher/scientist |
| Retired from any of the mentioned categories |
| I was a healthcare professional or researcher but switched careers |
| I prefer not to say |
| None of the above |
| Other (please specify) |
| **Q9. If you are not a healthcare professional or scientist, in which category does your profession best fit? If retired or unemployed, choose the one that most represents you.** |
| Architecture and engineering occupations |
| Arts, design, entertainment, sports, and media occupations |
| Business and financial occupations |
| Cleaning and maintenance occupations |
| Community and social service occupations |
| Computer and mathematical occupations |
| Construction and extraction occupations |
| Education, training and library occupations |
| Farming, fishing and forestry occupations |
| Food preparation and serving related occupations |
| Installation, maintenance and repair occupations |
| Legal occupations |
| Management occupations |
| Office and administrative support occupations |
| Personal care and service occupations |
| Production occupations |
| Protective service occupations |
| Sales and related occupations |
| Social science occupations |
| Transportation and materials moving occupations |
| Other |
| I prefer not to say |
| **Q10. Have you heard about the concept of "gut microbiota" or "gut microbiome" before?** |
| Yes |
| No |
| Rings a bell, but I am not sure |
| **Q11. If you have heard about the "gut microbiota" before, in which context? You can select different answers.** |
| In the media (TV, newspapers, radio, podcast...) |
| On social media platforms (Twitter, Facebook, Youtube...) |
| In the classroom / webinars / other type of formative studies |
| I work in the gut microbiota field / I collaborate with people that works in the gut microbiota field |
| A family member / friend has comment on that at some point |
| My medical doctor / nutritionist has mentioned it |
| I have never heard anything about the "gut microbiota"" |
| In a scientific activity / talk |
| I prefer not to say |
| Other (please specify) |
| **Q12. Have you ever searched for information on gut microbiota?** |
| Yes |
| No |
| I have found information about the gut microbiota while searching for other topics (gut diseases, special food, diet...) |
| **Q13. If you answered "yes" to the previous question, where did you search for information? You can select different answers.** |
| Blogs |
| Research articles in scientific journals |
| I asked a healthcare professional (medical doctor, nurse, nutritionist, etc) |
| Youtube (other social media) |
| I contacted a scientific communicator |
| I have not searched for information on gut microbiota |
| I prefer not to say |
| Other (please specify) |
| **Q14. Which of the following do you associate to "gut microbiota"? You can select different answers.** |
| Archaea |
| Bacteria |
| Epithelium |
| Faecal matter |
| Fungi |
| Mucus |
| Protozoa |
| Viruses |
| I do not associate any of these to "gut microbiota" |
| I do not know |
| I prefer not to say |
| **Q15. Which of the following factors do you believe determines gut microbiota composition? You can select different answers.** |
| Genetics |
| Place to live |
| Antibiotic consumption |
| Exercise |
| Profession |
| Diet |
| None of the above |
| I do not know what affects the composition of the gut microbiota |
| I do not know what the gut microbiota is |
| I prefer not to answer |
| **Q16. Can the gut microbiota affect your health?** |
| Yes |
| No |
| I do not know |
| **Q17. Can the gut microbiota affect your mood?** |
| Yes |
| No |
| I do not know |
| **Q18. Which of the following conditions do you believe could be related to some extent to gut microbiota alterations? You can select different answers.** |
| Autism |
| Anxiety |
| Breast cancer |
| Cardiovascular problems |
| Colon cancer |
| Depression |
| Diabetes |
| Inflammatory bowel disease (IBD), includes Chron's disease and ulcerative colitis |
| Irritable bowel syndrome (IBS) |
| Obesity |
| Parkinson's disease |
| All of the above |
| None of the above |
| I do not know |
| I prefer not to answer |
| **Q19. Which information can be obtained from a gut microbiota analysis?** |
| The presence of colorectal cancer |
| Inflammation levels in the gut in inflammatory bowel disease |
| The bacterial composition of the faeces |
| The bacteria, fungi, virus and protozoa present in the faeces |
| How gluten affects the gut mucosa in celiac people |
| Your dietary habits |
| All of the above |
| None of the above |
| I do not know |
| I prefer not to answer |
| **Q20. How do you order a gut microbiota analysis?** |
| I go to my family doctor (GP) |
| I go to the gastroenterologist |
| I order it to a company that performs these analyses |
| I do not know how to order a gut microbiota analysis |
| I prefer not to answer |
| **Q21. Which of the following therapies/techniques are you aware of? You can select different answers.** |
| Faecal transplant |
| Probiotics / Prebiotics / Synbiotics supplementation |
| Metabolomic profile |
| Patient's derived organoids / gut-on-a-chip |
| I have not heard about any of these therapies / techniques |
| I prefer not to say |
| **Q22. Would you be interested in learning more about the gut microbiota? (if you say "yes", no further contact will be made. This is purely for understanding the public´s interest level on the topic)** |
| Yes |
| No |
| **Q23. Which medium do you think you would engage with to learn about gut microbiota? You can select different options.** |
| Books and press articles |
| E-learning |
| Webinars |
| Videos |
| Infographics |
| Videos in social media platforms (Instagram, Tik Tok, Facebook, Twitch, etc) |
| Podcast |
| None of the above |
| I am not interested in the topic |
| Other (please specify) |
| **Q24. For healthcare professionals. How often do you receive patients referring to persistent abdominal pain and feeding problems that do not improve with dietary changes?** |
| Very frequently |
| Often |
| Sometimes |
| Rarely |
| Never |
| I am not a healthcare professional |
| I do not receive patients |
| I prefer to not answer |
| **Q25. For healthcare professionals. Have you recommended a gut microbiota analysis for a patient?** |
| Yes |
| No |
| I do not receive patients |
| I am not a healthcare professional |
| **Q26. For healthcare professionals. If you have recommended / ordered a gut microbiota analysis, which strategy have you followed?** |
| The analysis are performed in the same centre / subsidiary centre |
| The patients were part of a clinical study |
| The analyses are performed in a research centre that we collaborate with |
| A private company that offers these services |
| I have never recommended / ordered a gut microbiota analysis |
| I do not know how to order a gut analysis |
| I prefer not to say |
| I am not a healthcare professional |
| **Q27. For healthcare professionals. If you have never recommended gut microbiota analysis, what are the barriers to you doing so? You can select different answers.** |
| We do not have the facilities or resources necessaries to conduct these studies |
| I prefer conducting other type of analyses that I believe could be more informative / relevant for the patient's diagnostics |
| They are expensive studies |
| I do not think that the gut microbiota is involved in the clinical profiles that I work with |
| I did not consider it |
| I do not know how to interpret the results of this type of analyses |
| I do not think that the information that can be obtained would be relevant |
| I prefer not to say |
| I have recommended gut microbiota analysis |
| I am not a healthcare professional |
| I do not receive patients |


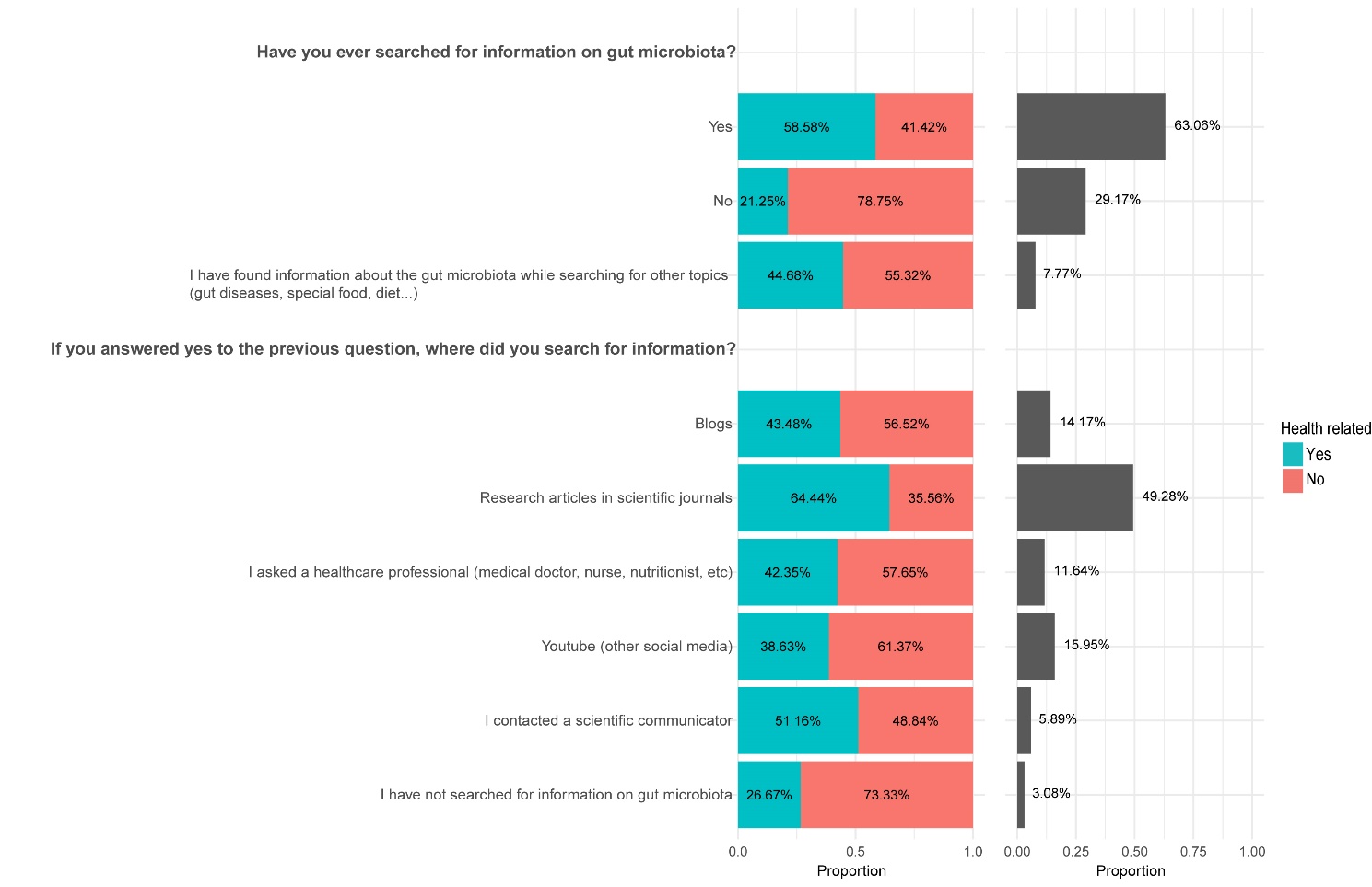


**Supplementary Figure S1.** **Active information source for the gut microbiota.** Information sources on gut microbiota that the participants had previously used involving active and self-initiated searches. A total of 1210 participants answered the query “Have you ever searched information on gut microbiota?” while a total of 925 participants responded to the query “If you answered yes to the previous section, where did you search for information?”.
